# Vascular Disease Patient Information Page: A guide for patients with newly diagnosed deep vein thrombosis or pulmonary embolism

**DOI:** 10.1177/1358863X231154756

**Published:** 2023-07-04

**Authors:** Cassiopeia Frank, Elizabeth V Ratchford, Stephan Moll

**Affiliations:** 1 Department of Medicine, Division of Hematology, University of North Carolina Chapel Hill School of Medicine, Chapel Hill, NC, USA; 2 Johns Hopkins Center for Vascular Medicine, Johns Hopkins University School of Medicine, Baltimore, MD, USA

**Keywords:** anticoagulation, deep vein thrombosis (DVT), pulmonary embolism (PE), venous thromboembolism (VTE)

## What is venous thromboembolism (VTE)?

Blood clots in the deep veins and/or lungs are referred to as venous thromboembolism (VTE). Deep vein thrombosis (DVT) is when a blood clot forms in deep veins, which can be located anywhere in the body but most often in the legs. A complication that can occur from a DVT is a pulmonary embolism (PE), which is when a blood clot breaks off and travels to the lungs. Although a PE can heal completely, it can also have severe consequences, including death. DVTs and PEs are almost always treated with blood thinners (anticoagulants). Common terms and abbreviations are explained in Table 1.

**Table 1.** Dictionary of relevant terms.

Abbreviation	Term	Definition
DVT	Deep vein thrombosis	A clot in a deep vein, most commonly in the legs
PE	Pulmonary embolism	A clot that has traveled to the lungs
VTE	Venous thromboembolism	DVT or PE
CTEPH	Chronic thromboembolic pulmonary hypertension	Increased pressure in the vessels in the lung and stress on the heart
PTS	Post-thrombotic syndrome	A term used to describe long-term symptoms in the leg following a DVT
DOAC	Direct oral anticoagulant	A type of blood-thinning medication
–	Anticoagulant	Blood-thinning medication
–	Thrombophilia	Condition that makes one more likely to develop blood clots

Dash (–): not applicable.

## How and where do blood clots form?

**Figure 1.**
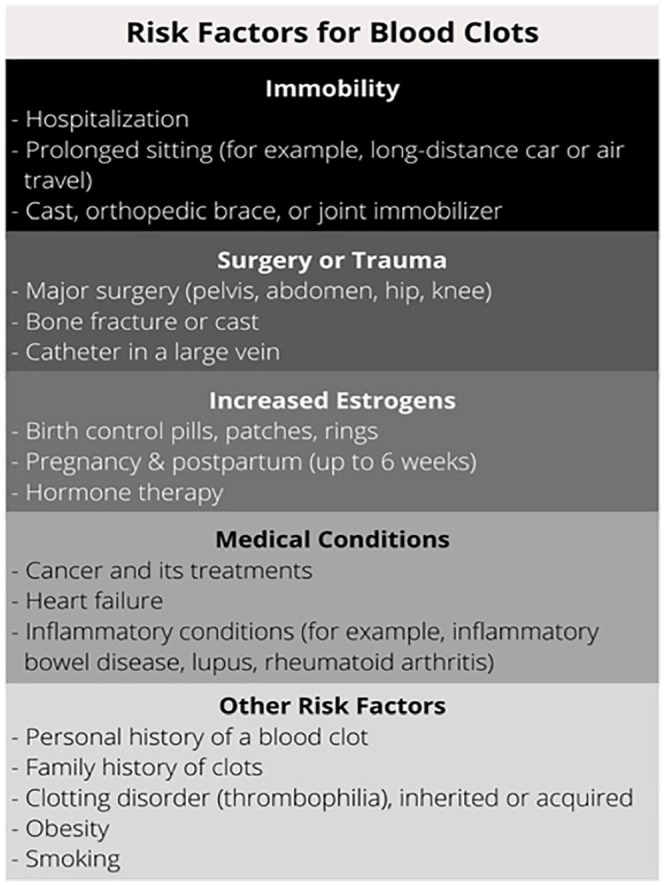
Summary of risk factors for blood clots (deep vein thrombosis and pulmonary embolism).

Blood clots can happen for many reasons, but generally they develop because of one or more risk factors listed in Figure 1. Often, several of these risk factors contribute to the development of a clot. Sometimes blood clots can happen for reasons that are never identified; these are called ‘unprovoked’ (i.e., idiopathic or unexplained) blood clots.

**Figure 2.**
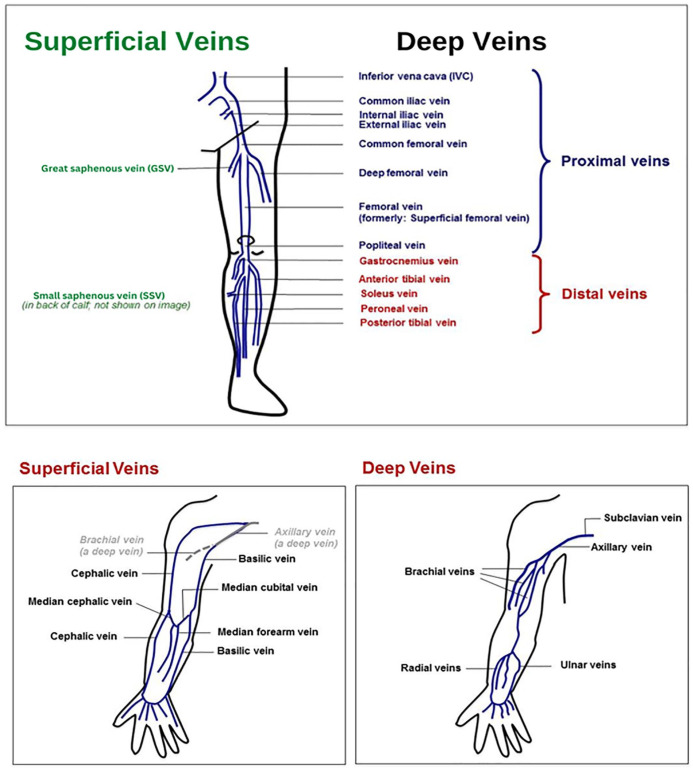
Superficial and deep veins of the leg and arm. A health care provider can mark the location of the clot to help the patient understand which vein(s) are involved.

Blood clots can be found in deep veins (deep inside the body; more serious) or superficial veins (closer to the skin). They can also be closer to the large central veins (proximal) or farther away (distal) in smaller veins. Figure 2 shows some of the common superficial and deep veins in the arms and legs where blood clots may form. The location of the blood clot is an important factor in assessing the risk of complications and determining how long to treat with blood thinners.

## What to expect after DVT or PE is diagnosed

The next steps after diagnosis of DVT and/or PE vary based on the medical history, age of the patient, and the circumstances surrounding the clot.

**Figure 3.**
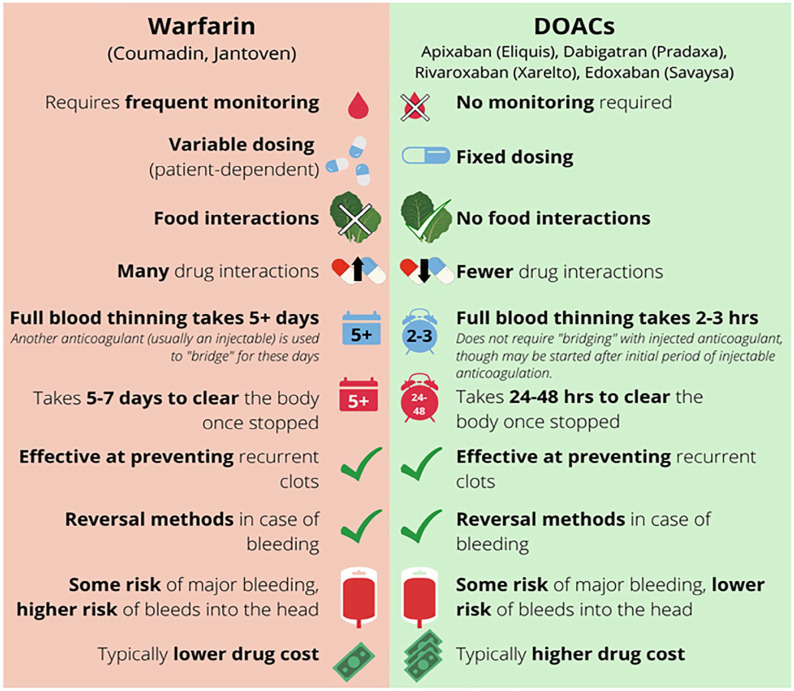
The similarities and differences between the two types of commonly prescribed blood thinners: warfarin and direct oral anticoagulants (DOACs).

Blood tests to check for anemia and kidney function help determine which blood thinner medications (anticoagulants) might be appropriate and identify potential risks for taking blood thinners. The choice of blood thinner depends on several factors. The available medications include pills taken by mouth as well as injectable drugs. Two commonly prescribed blood thinners are warfarin and direct oral anticoagulants (DOACs). For more information, see the Patient Information Page on DOACs.^1^ Further treatment considerations include kidney function, bleeding risks, and cost. A comparison of available oral blood thinners (warfarin vs DOACs) is shown in Figure 3.

In addition, routine age-appropriate cancer screening (e.g., mammogram or colonoscopy) is generally recommended. The decision to pursue thrombophilia testing^2^ will depend on several factors including patient preference, impact on treatment plan, and cost. Depending on results of thrombophilia testing, familial testing for a clotting disorder is sometimes considered.

## What kind of specialist takes care of patients with blood clots?

The type of doctor needed depends on the patient and circumstances surrounding the blood clot. A primary care physician can help decide what specialty care might be needed, which may include a vascular medicine specialist and/or a hematologist. In rare cases, patients with blood clots with severe symptoms may be referred to interventional vascular specialists, including interventional cardiologists, interventional radiologists, or vascular surgeons. If available, a visit to a Thrombosis Clinic or Vascular Clinic may be recommended. Additionally, if warfarin is prescribed, a specialized Anticoagulation Clinic may be beneficial.

## How are DVT and PE treated?

Blood thinner medications (anticoagulants) are typically prescribed for the treatment of a DVT or PE. These medications increase the time it takes for blood to clot and helps to prevent new clots from forming and existing clots from growing. Anticoagulation therapy allows the body to dissolve the clot over time while preventing a new or worsening blood clot.

**Figure 4.**
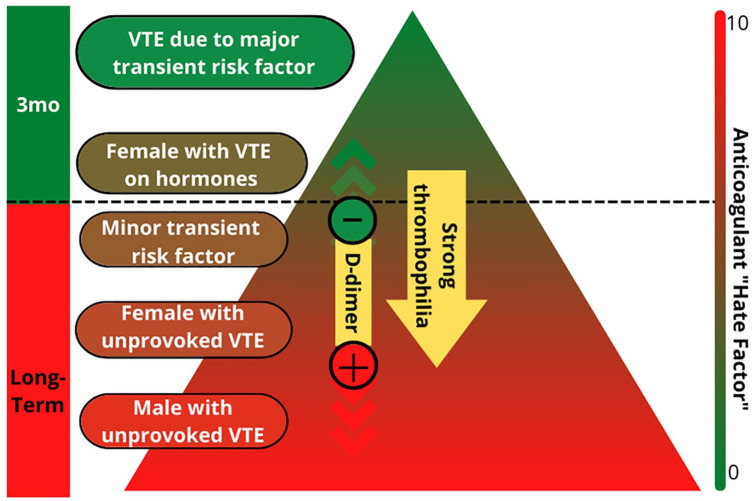
Recurrence triangle. The recurrence triangle is a tool used to help determine how long to continue blood thinner. The triangle considers why the initial venous thromboembolism (VTE) occurred and the sex of the patient. The green top of the triangle indicates a low risk of another clot if blood thinner is stopped; for patients who are in this part of the triangle, short-term treatment (3–6 months) is recommended. If a patient is in the red zone of the triangle, risk for recurrence is high if blood thinner is stopped, and long-term treatment is recommended. Blood tests, including a ‘D-dimer’, or tests for a strong clotting disorder (thrombophilia), may also be used, particularly in patients with an intermediate risk of recurrence, to help determine whether to stop or continue blood thinners. On the right side, a patient’s preference for stopping or continuing the blood thinner is taken into consideration.

The duration of anticoagulation therapy depends on several factors. The first determining factor is the location of the clot. A superficial or distal clot may not require an anticoagulant at all or may require one for only a few weeks. A proximal clot or PE will typically require at least 3 months of treatment. After that, the decision to continue treatment depends on factors that contributed to formation of the blood clot^3^ (Figure 4). Clots that develop due to transient risk factors (provoked blood clots) may be treated for a short period of time (typically 3–6 months). Clots that develop for unclear reasons (unprovoked) or for reasons that are likely to persist (e.g., cancer, chronic immobility, a strong clotting disorder) may require long-term treatment to prevent recurrence. Recurring blood clots are almost always treated with long-term anticoagulation therapy; however, that decision also depends on what triggered previous clots. Other factors to consider include risk of bleeding and personal preference on taking blood thinners (i.e., ‘anticoagulant hate factor’) (Figure 4). If long-term blood thinners are prescribed, regular follow-up (e.g., once per year) is recommended to reassess options based on how things are going with the medication, discuss new research or drugs that may change the treatment plan, and check routine lab work.

## Are there any physical limitations during or after treatment for DVT or PE?

Rest and recovery are important after any illness or injury, but exercise and physical activity are encouraged following diagnosis of DVT or PE as long as it feels comfortable. Pushing through despite pain or shortness of breath will not speed up recovery and should be avoided. However, if one feels well enough to exercise and/or return to work, it is generally safe to do so. It is important to remember that although exercise is safe from a DVT/PE perspective, high-risk physical activities where bodily injury is possible should be avoided while on blood thinners. People taking blood thinners should consider carrying a medical alert bracelet or tag, which can alert medical professionals in case of an emergency.

Regarding travel, there is no clear guidance regarding the safety of plane travel immediately after a blood clot. It may be best to avoid flying in the first 4 weeks, but this is not an absolute recommendation. It is important to discuss the risks and benefits with your doctor. Massage of the extremity with the DVT is best avoided in the first few weeks of a DVT due to concerns that a clot might break off and become a PE with deep massage. Massages at a later date and while taking a blood thinner are not prohibited, but more forceful deep tissue massage that could cause bruising or bleeding should be avoided on blood thinners.

## When will the symptoms improve?

Symptoms, including shortness of breath, chest pain, rapid heart rate, and leg pain, swelling, or redness, will gradually improve and often fully resolve. The highest risk period for serious complications is in the first few days, when there is a risk of a clot breaking off from the DVT and traveling to the lungs, causing a PE. Most people with DVT and PE will notice improvement fairly quickly (within days to weeks) after starting blood thinner medication and will have complete resolution of symptoms by 3 months. In some cases, there are longer-term symptoms, as noted below.

## What are the long-term risks after a blood clot?

Although most patients will recover completely from a DVT/PE, some long-term symptoms may persist. After a DVT or PE, post-thrombotic syndrome (PTS) or post-PE syndrome may develop. If damage to the lung is severe, chronic thromboembolic pulmonary hypertension (CTEPH) may occur.

### Post-thrombotic syndrome (PTS)

About 40% of people who have a DVT will develop some long-term (chronic) symptoms in the affected leg. PTS can be very mild and not bothersome but for some patients it can be severe and disabling. Symptoms of PTS include:

– Chronic leg swelling;
– Chronic pain/pressure, heaviness, tightness, or leg fatigue;
– Skin hardening, dryness, or itching;
– Dark pigmentation;
– Visible spider veins; and
– Skin ulcers can develop from skin breakdown in affected areas in the leg in severe cases.

Symptoms of PTS can be improved with graduated compression stockings, which are tight at the ankle and less tight toward the knee or thigh. These stockings can help push fluid out of the lower portion of the leg, relieving swelling and pain. They come in different levels of tightness, measured in millimeters of mercury (mmHg) like a blood pressure cuff. For more information, see the Patient Information Page on compression therapy.^4^ Compression therapy may also relieve pain and swelling after the initial diagnosis of DVT.

### Post-PE syndrome

Shortness of breath following a PE may persist even without evidence of extensive permanent lung damage or pulmonary hypertension. Patients may also have intermittent chest pain, pressure, or discomfort, particularly when taking a deep breath. These symptoms can be a mild nuisance or may impact quality of life if severe. Testing for post-PE syndrome is similar to CTEPH.

### Chronic thromboembolic pulmonary hypertension (CTEPH)

When blood clots in the lungs do not resolve, chronic lung damage can occur, increasing the pressure in the vessels in the lung and the stress on the heart. This condition is called pulmonary hypertension. The most common symptom of CTEPH is unresolved or worsening shortness of breath, especially with exertion. If CTEPH is suspected, next steps may include a heart echo (echocardiogram), a ventilation/perfusion nuclear medicine lung scan, and/or referral to a pulmonologist (lung doctor).

## Is it normal to be anxious or depressed after DVT or PE diagnosis?

A diagnosis of DVT or PE brings many challenges, both physical and psychological. Immediately after diagnosis, a patient may be dealing with physical pain, trying to understand why the clot happened, and adjusting to the lifestyle impact of taking an anticoagulant. It is normal to feel shocked, anxious, and fearful after the diagnosis of a blood clot.

Temporary feelings of anxiety or depressed mood can occur in the first few weeks, but the fear of a future clot recurrence can produce ongoing anxiety. Support groups may be helpful (Table 2). If these feelings do not improve or are accompanied by a withdrawal from activities or increased negative thoughts and tearfulness, then treatment by a mental health professional may be needed. Resources available for patients with newly diagnosed thrombosis are outlined in Table 2.

**Table 2.** Resources available for patients with newly diagnosed thrombosis.

Organization and website	Type of resource
**National Blood Clot Alliance**	Patient information resource and national advocacy organization
www.stoptheclot.org	
**North American Thrombosis Forum**	Patient information resource
https://thrombosis.org/patients/	
**Anticoagulation Forum**	Listing of anticoagulation clinics
https://acforum.org/web/clinics.php	

## Summary

Blood clots in the extremities (deep vein thrombosis) or lungs (pulmonary embolism) can be serious and potentially life-threatening. Treatment using blood-thinning medication is effective for managing both types of blood clots. The decision regarding what type of blood thinner to use and how long to prescribe it is based on several factors, including why the blood clot developed, the personal and family medical history of the patient, and the bleeding risk. In some cases, patients stay on blood thinners long-term to prevent recurrent blood clots.

The ‘Vascular Disease Patient Information Page’ is a regular feature of *Vascular Medicine*. All articles in the collection are available for free online at http://journals.sagepub.com/vmjpatientpage.

The Vascular Disease Patient Information Page is provided for educational purposes only and is not a substitute for medical advice.
